# Host Protective Immune Responses against Influenza A Virus Infection

**DOI:** 10.3390/v12050504

**Published:** 2020-05-03

**Authors:** Hi Eun Jung, Heung Kyu Lee

**Affiliations:** 1 Graduate School of Medical Science and Engineering, Korea Advanced Institute of Science and Technology (KAIST), Daejeon 34141, Korea; 2 KAIST Institute for Health Science and Technology, KAIST, Daejeon 34141, Korea

**Keywords:** influenza virus, anti-influenza immune responses

## Abstract

Influenza viruses cause infectious respiratory disease characterized by fever, myalgia, and congestion, ranging in severity from mild to life-threating. Although enormous efforts have aimed to prevent and treat influenza infections, seasonal and pandemic influenza outbreaks remain a major public health concern. This is largely because influenza viruses rapidly undergo genetic mutations that restrict the long-lasting efficacy of vaccine-induced immune responses and therapeutic regimens. In this review, we discuss the virological features of influenza A viruses and provide an overview of current knowledge of the innate sensing of invading influenza viruses and the protective immune responses in the host.

## 1. Introduction

The influenza virus, or ‘flu’, causes an acute respiratory disease that results in moderate to severe symptoms including fever, runny nose, cough, muscle pain, headaches, and sometimes even death. Each year, seasonal influenza strains spread across the globe, causing significant economic and clinical burdens. As a result, considerable resources have been devoted to preventing and treating flu infections. Vaccination is currently the most effective method for prevention of the flu. The World Health Organization releases a recommendation for the composition of the seasonal influenza vaccine each year on the basis of strains that are predicted to circulate [1]. Although vaccination results in protective immune responses against the surface antigens of influenza, continuous genetic mutations allow the virus to eventually bypass vaccine-induced protection. To date, three classes of anti-influenza drugs have been approved by the Food and Drug Administration (FDA). These drugs target several distinct steps of the viral replication cycle to inhibit influenza propagation in hosts. Matrix 2 (M2) inhibitors block the release of viral ribonucleoproteins (vRNPs) into the cytosol, and neuraminidase (NA) inhibitors prevent viruses from budding out of host cells. The cap-dependent endonuclease inhibitor, which received FDA approval for the treatment of influenza in 2018 [2,3], targets the influenza polymerase acidic (PA) endonuclease to prevent viral replication [4]. While these drugs are currently effective, they are only capable of reducing symptoms by a few days. Additionally, constant mutations in the influenza virus can result in resistance, thus reducing therapeutic efficacy.

Throughout history, several influenza pandemics have emerged as a result of cross-species transmission, resulting in millions of deaths. For example, a novel H1N1 strain was generated in 2009 after a reassortment event among H1N1 influenza subtypes in swine [5], and that virus subsequently spread across the world to cause 105,700–395,600 deaths [6]. Seasonal epidemic influenza, which is marked by antigenic drift, is particularly dangerous for specific groups such as pregnant women, young children, the elderly, and patients with comorbidities. Because seasonal influenza mutations and cross-species events are relatively unpredictable, it is important to understand the factors that influence successful defense against influenza infections.

In this review, we describe the virological characteristics of influenza A viruses that facilitate viral propagation in host cells and the mechanisms by which host cells recognize invading influenza viruses and produce a protective response against infection.

## 2. Virological Features of Influenza A Virus

The influenza virus is a respiratory pathogen that causes acute febrile disease called influenza or flu. It belongs to the *Orthomyxoviridae* family and can infect diverse host species including humans, pigs, and birds. The influenza viruses that cause disease in humans are classified into three types—influenza type A, B, and C—primarily based on antigenic differences in their NP and M proteins [7,8]. The novel influenza D virus, which was first identified in 2011 [9], infects animals such as cattle and pigs [10,11]. However, it remains unclear whether influenza D virus (IDV) can cause disease in humans. While IDV infection in humans has not yet been reported, IDV specific antibodies have been detected in human serum samples from cattle-exposed workers, indicating that this virus has the potential to elicit an immune response in humans [10,12]. 

**Figure 1:**
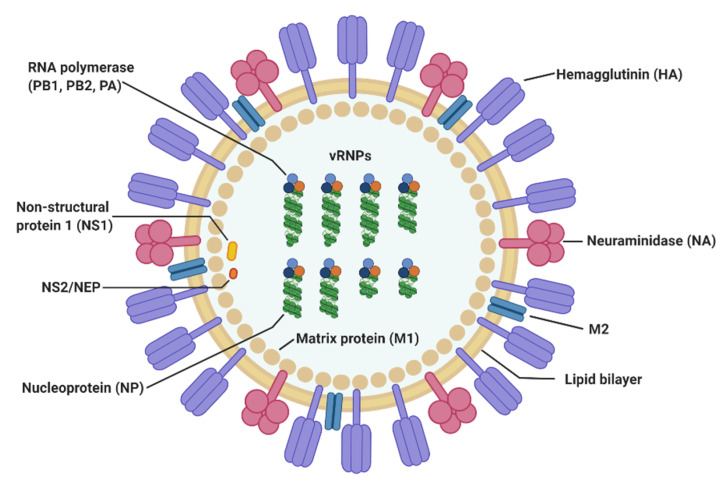
The structure of influenza A virus. IAV is a negative-stranded RNA virus belonging to the *Orthomyxoviridae* family. The IAV genome is divided into eight segments that encode 11 viral proteins in total (HA, NA, M1, M2, NP, NS1, NS2, PA, PB1, PB2, and PB1-F2). The viral envelope of IAV contains the transmembrane proteins HA, NA, and M2.

Influenza A and B viruses are the most common causes of seasonal flu epidemics in humans [13]. Influenza B virus (IBV), which generally circulates later in the season, is responsible for 15–30% of total influenza infections [14]. While the disease severity due to both types is comparable [15,16,17], IBV does not cause pandemics. In contrast, strains of influenza A virus (IAV) are often responsible for seasonal influenza epidemics and pandemic outbreaks due to frequent genetic mutations and inter-subtype reassortment [18]. The IAV virion is covered by a lipid-protein envelope containing the transmembrane proteins hemagglutinin (HA), NA, and M2 (Figure 1). The genome of IAV consists of single-stranded, negative-sense RNA that is split into eight segments encoding a total of 11 viral proteins: HA, NA, M1, M2, NP, non-structural protein 1 (NS1), non-structural protein 2 (NS2), PA, polymerase basic protein 1 (PB1), polymerase basic protein 2 (PB2), and polymerase basic protein 1-F2 (PB1-F2). Each segment forms a vRNP complex that is comprised of viral RNA and NP, which then combines with the RNA polymerase PB1-PB2-PA complex. The M1 protein, which exists only on the inside of the viral envelope, interacts with vRNPs [19]. The viral envelope of IAV consists of a lipid bilayer with viral transmembrane proteins called HA, NA and M2. HA recognizes the sialic acid (SIA) receptors expressed on the surface of host cells in the respiratory tract and is responsible for viral attachment and entry. M2 is a proton-selective ion channel that is activated by the drop in pH that occurs after virion endocytosis and endosomal acidification. It mediates the acidification of the viral core via the introduction of protons and results in the release of vRNP into the host cell’s cytoplasm. NA is essential for the spread of newly synthesized viruses from host cells. It cleaves the SIA residues of glycoproteins to allow viral release and to prevent aggregation of individual virions [20]. At present, 18 subtypes of HA and 11 subtypes of NA have been documented [21], and IAVs are divided into subtypes based on the combination of HA and NA. Antigenic drift and antigenic shift, the primary mechanisms behind the antigenic variation of the influenza virus, occur in both HA and NA. The accumulation of random mutations (antigenic drift) in HA and NA, and new combinations of sequences from two or more flu strains (antigenic shift) can generate novel viruses that are different from pre-existing subtypes, and are capable of bypassing pre-existing adaptive immunity, to cause influenza pandemics [22,23,24,25].

To achieve successful infection, the influenza virus must first pass through the respiratory mucus layer that forms a primary physical barrier. Mucus in the respiratory tract contains sialylated glycoproteins. Previous research has demonstrated that sialylated decoy receptors expressed in the airway mucus protect the underlying cells from infection by inhibiting viral entry [26]. However, the influenza virus cleaves sialylated mucins using NA, which disables the inhibitory functions of the mucus, thus allowing penetration into the mucus layer. Next, virions bind to the SIA-containing receptor using HA and enter the host cell via receptor-mediated endocytosis. However, SIA-independent influenza infection has also been reported [27,28]. C-type lectin receptors are thought to act as alternative receptors that allow infectious entry of the influenza virus in a manner independent of SIA. The macrophage’s galactose-type lectin and mannose receptors play important roles in influenza infection [29,30,31]. DC-SIGN (DC209) and L-SIGN (CD209L) have been identified as influenza attachment receptors [32,33,34,35], and the blocking of DC-SIGN decreases the rate at which the virus infects dendritic cells (DCs) isolated from peripheral blood mononuclear cells [32]. These results suggest that C-type lectin receptors can act as specific receptors for IAV infection. M2 proton channels mediate endosomal acidification, resulting in the fusion of viral and endosomal membranes and the release of vRNPs into the cytosol [22,36,37,38,39,40]. Released vRNPs are then translocated into the host nucleus via nuclear pore complexes, where they transcribe and replicate viral RNA (vRNA). RNA-dependent RNA polymerase (RdRP) is responsible for both transcription and replication of the viral RNA genome. The influenza RdRP protein consists of three subunits: PB1, PB2, and PA. Specifically, a “cap-snatching” mechanism is required for the transcription of viral mRNA. PB2 recognizes the 5′ cap of host pre-mRNA, and PA cleaves host mRNA to generate 5′-capped RNA fragments that are then used as primers to initiate viral mRNA transcription [41,42]. PB1 carries out viral mRNA synthesis using these short fragments [43,44]. In contrast, vRNA replication, which occurs through a complementary RNA intermediate, is primer-independent [45,46]. NS1 plays an important role in the inhibition of host antiviral immune responses. Previous studies have shown that NS1 suppresses the expression of host mRNAs that enable interferon (IFN)-induced antiviral phenotypes [47,48,49]. Further, NS1 restricts nucleocytoplasmic export of host mRNA by targeting nuclear RNA export factor 1–nuclear transport factor 2-related export protein 1 [50,51,52], inhibits caspase-1 activation as well as the production of interleukin-1β (IL-1β) [53], and disturbs the RIG-I signaling pathway [54,55,56,57]. 

NS2, also known as the nuclear export protein, transports newly synthesized RNPs out of the nucleus after amplification [58,59]. Further, it has been suggested that NS2 is important for efficient influenza virion formation and budding due to its interactions with the F1Fo-ATPase [60]. The IAV virulence protein PB1-F2 contributes to inflammatory responses and flu-induced pathogenesis through activation of the NLRP3-inflammasome [61]. 

**Figure 2:**
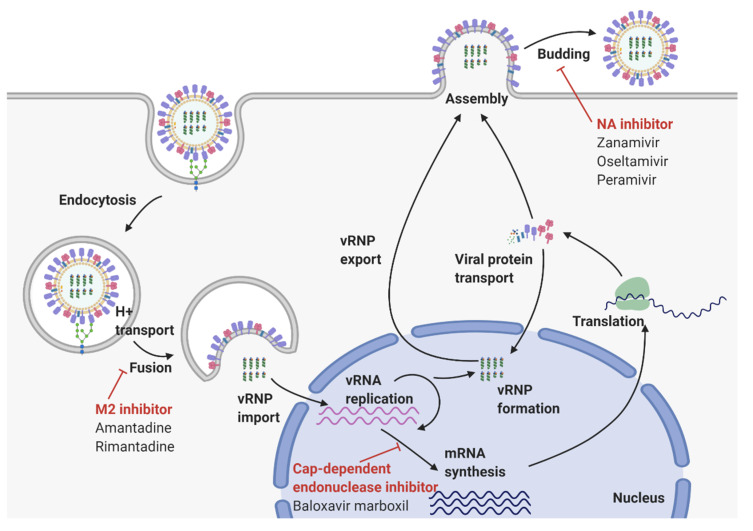
Influenza A replication cycle. The virus recognizes specific receptors expressed on the host cell surface using HA and enters cells via endocytosis. After fusion of the viral envelope and endosomal membrane, vRNPs are released into the cytoplasm and translocate into the nucleus to initiate replication. RdRP is responsible for both viral mRNA transcription and vRNA replication. Newly synthesized vRNPs are exported to the cytoplasm, and assembly of progeny virions occurs near the plasma membrane. NA facilitates budding of new virions from host cells. Anti-influenza drugs inhibit various steps of the influenza virus replication cycle.

Therapeutics targeting various stages of the influenza virus’ replication cycle have been developed. There are three classes of FDA-approved anti-influenza drugs: M2 inhibitors (Amantadine, Rimantadine), NA inhibitors (Oseltamivir, Peramivir, Zanamivir), and a cap-snatching inhibitor (Baloxavir marboxil). M2 inhibitors block the release of vRNPs into the cytosol. However, most circulating viruses are now resistant to the existing M2 blockers, and they are no longer recommended for treatment or prophylaxis of influenza. NA inhibitors prevent virions from budding from host cells, and the cap-dependent endonuclease inhibitor targets influenza polymerase to prevent viral replication. While these drugs are currently effective, constant changes in the viral genome cause drug resistance and reduce therapeutic efficacy (Figure 2).

## 3. Innate Sensors Recognizing the Influenza Virus

After entering the host, the influenza virus infects bronchiolar-alveolar epithelial cells lining the respiratory tract and replicates to spread throughout the airways [62]. Influenza can also spread to immune cells such as macrophages and DCs in the lungs [23]. The innate immune system is the first protective barrier against invading organisms and is responsible for the initiation of adaptive immune responses. To detect invading pathogens, cells of the innate immune system have pattern recognition receptors (PRRs) that recognize pathogen-associated molecular patterns (PAMPs) and initiate innate immune responses. Recognition of the influenza virus is mediated by several types of PRRs, including Toll-like receptors (TLRs), retinoic acid-inducible gene I (RIG-I), nucleotide-binding oligomerization domain (NOD)-like receptor family pyrin domain-containing 3 (NLRP3), and the cyclic guanosine monophosphate-adenosine monophosphate synthase (cGAS) pathway. Activation of PRRs results in the expression and secretion of proinflammatory cytokines and type I IFNs, which establish an antiviral immune microenvironment.

### 3.1. TLRs

TLR3 is the receptor for double-stranded RNA (dsRNA). Although IAV does not produce dsRNA during replication [63,64], TLR3 activation can occur during influenza infection [65]. However, the specific TLR3 ligand that is produced during IAV infection has not yet been determined. The resulting TLR3 signaling process induces production of proinflammatory cytokines and type I IFN via Toll/IL-1 receptor-domain-containing adapter-inducing interferon-β (TRIF)-mediated activation of IFN-regulatory factor 3 (IRF3) and nuclear factor-κB (NF-κB) [66]. Previous research has shown that TLR3 signaling promotes the production of proinflammatory cytokines in the bronchoalveolar airspace and contributes to viral pathology during H3N2 infection, and viral burden is increased in the lungs of TLR3 knockout mice [67]. However, TLR3 signaling does not seem important for adaptive immune responses against influenza, because T and B cell responses are unaffected by TLR3 deficiency [68].

TLR7, the receptor for single-stranded RNA (ssRNA), is involved in the recognition of the influenza virus [69]. Plasmacytoid DCs (pDCs) express TLR7 in their endosomes, implying that the detection of ssRNA by TLR7 can occur prior to actual viral replication in pDCs [70]. TLR7 signaling induces the activation of IRF7 and NF-κB via the MyD88-mediated pathway, which initiates the expression of proinflammatory cytokines and type I IFNs. Due to conflicting results from previous studies, it remains unclear whether TLR7-MyD88 signaling is necessary for the protection against influenza infection. However, TLR7 deficiency results in attenuated production of type I IFN by pDCs [69], reduced IL-1β secretion from bone marrow-derived DCs [71], and impaired activation and production of IFN-γ by lung natural killer cells (NK cells) during infection [72], suggesting that TLR7 is responsible for efficient innate immune responses against the influenza virus. Indeed, prior evidence has supported the function of TLR7-MyD88 signaling in humoral responses [68,73,74,75,76].

### 3.2. RIG-I Signaling Pathway

RIG-I is a cytosolic sensor of short viral 5′-triphosphorylated ssRNA or dsRNA. The 5’-triphosphate-containing vRNA that is produced during viral replication is recognized by RIG-I. RIG-I uses its helicase domain to detect the 5’-triphosphate ssRNA and subsequently recruits the mitochondrial antiviral signaling protein (MAVS, also known as IPS-1) using caspase-recruitment domains (CARD). Subsequent RIG-I-MAVS signaling induces the activation of IRF3/7 and NF-κB, which then initiates the production of proinflammatory cytokines and type I IFNs. Although replication of the influenza virus occurs in the nucleus, the RIG-I signaling pathway is activated soon after infection. A recent study revealed that RIG-I is located in antiviral stress granules that consist of vRNA and proteins produced by protein kinase R (PKR) [77]. Interestingly, this stress granule serves as the site for detection of viral ssRNA by RIG-I. Of note, the influenza virus actually prevents the formation of these stress granules by inhibiting PKR activity in a manner dependent on functional NS1 protein [78,79]. Taken together, the influenza virus NS1 protein disrupts RIG-I signaling, thus suppressing IFN-α/β production [54,55,56,57,64]. However, RIG-I-MAVS signaling does not seem to play a critical role in the defense against viral infection; survival and adaptive immune responses against the influenza virus were unchanged in MAVS-deficient mice relative to wild-type controls [76]. 

### 3.3. Inflammasomes

NLRP3 is an intracellular sensor belonging to the group of NOD-like receptors (NLRs) and is an intracellular sensor of PAMPs and endogenous danger signals. When stimulated by cellular stress or intracellular infection, it activates the NLRP3 inflammasome, a multimeric complex that initiates inflammatory cell death. NLRP3 is primarily expressed by macrophages, monocytes, neutrophils, and DCs [80]. Specifically, the activation of NLRP3 results in the recruitment of apoptosis-associated speck-like protein containing a carboxy-terminal CARD (ASC), the adaptor protein that connects NLRs and capase-1, and assembles a multimeric cytoplasmic complex called the inflammasome. The inflammasome mediates production of the proinflammatory cytokines IL-1β and IL-18. Several influenza virus proteins are associated with the activation of the NLRP3 inflammasome. For example, M2 protein, the proton channel of the influenza virus, activates the formation of the NLRP3 inflammasome [71]. Localization of M2 in the Golgi apparatus is essential for inflammasome activation. Interestingly, influenza virus NS1 proteins inhibit the secretion of IL-1β through interactions with NLRP3 [53,81]. The RNA-binding domain and TRIM25-binding domain of NS1 are associated with NS1-induced NLRP3 suppression. Further, the accumulation of PB1-F2 in the phagosome activates the NLRP3 inflammasome in macrophages [61]. PB1-F2 peptides trigger IL-1β production, but knockdown of NLRP3 prevent the PB1-F2-induced secretion of IL-1β. These findings suggest that PB1-F2 facilitates activation of the NLRP3 inflammasome. However, another study found that mitochondrially targeted PB1-F2 impairs innate immune responses by suppressing RIG-I signaling and NLRP3 inflammasome activation [82]. PB1-F2 translocates to the mitochondria in a manner dependent on Tom40 channels and causes defects in mitochondrial dynamics, resulting in the inhibition of RIG-I and NLRP3-mediated innate immune responses. Further studies are required to elucidate the interactions between PB1-F2 and activation of the inflammasome.

Several lines of evidence support a crucial role for the inflammasome in cellular defense against influenza virus infection. Inflammasome-deficient mice have impaired recruitment of monocytes and granulocytes to the lung and suffer from significantly increased disease severity compared to control mice. Although inflammasome deficiency did not affect viral clearance within the first 6 days after infection, late viral clearance (7–10 days post-infection) was significantly decreased by inflammasome deficiency [83,84,85]. Prior work has shown that the inflammasome plays a critical role in the activation of T and B cell responses against the influenza virus during low dose infection; deficiency of the inflammasome adaptor protein ASC and the downstream protein caspase-1 lead to impaired adaptive immune responses [85].

### 3.4. cGAS-STING Pathway

cGAS is a well-known cytosolic DNA sensor that mediates cytosolic DNA-induced immune responses though its downstream signaling molecule, stimulator of interferon genes (STING) [86]. Accumulation of cytosolic DNA triggers activation of cGAS-STING, resulting in the production of type I IFN and inflammatory cytokines through activation of IRF3 and NF-κB. Self-DNA, mitochondrial DNA (mtDNA), viral DNA, and bacterial DNAs are known ligands of cGAS [86,87,88,89,90,91]. It is well established that the cGAS-STING pathway plays an important role in the defense against DNA viruses including the herpes simplex virus [92]. Interestingly, a recent study has revealed that infection with influenza, an RNA virus, also activates the cGAS-STING pathway [93]. After influenza virus infection, the M2 protein promotes the release of mtDNA into the cytosol, where it stimulates the cGAS pathway. Interestingly, the influenza virus NS1 protein appears to associate with mtDNA and inhibit mtDNA-induced expression of IFN-β. In addition, the relative expression of IFN-β mRNA in flu-infected lung tissue is reduced in cGAS- or STING-deficient mice. However, it is still unclear whether the cGAS-STING pathway is crucial for host survival after influenza virus infection.

## 4. Innate Effector Cell Types in the Response to Influenza Infection

During influenza virus infections, innate signaling and adaptive immune responses both play important roles in protecting against the virus and achieving viral clearance. At the site of infection, infected cells produce chemokines and cytokines such as type I IFNs, IL-6, IL-8, TNF-α, CCL2 (MCP-1), RANTES, and MIP-1α that recruit immune cells including NK cells, neutrophils, macrophages and DCs to the site of infection where they initiate the innate immune response [94,95,96]. These immune cells are responsible for both protection and immunopathology following influenza virus infection.

### 4.1. Natural Killer Cells, Neutrophils, and Macrophages

Innate cells are initially recruited to the site of infection from the systemic circulation by the proinflammatory cytokines that are released by infected lung epithelial cells. NK cells recognize HA on influenza-infected cells via NKp44 and NKp46 receptors [97,98,99] or detect antibody-coated infected cells via CD16 [100]. Then, activated NK cells lyse the infected cells by secreting perforin and granzyme [101,102]. Defects in NK cells lead to increased susceptibility to influenza infection and uncontrolled viral growth in the lungs [103,104]. However, excessive NK cell-induced inflammation can also contribute to pathology following high dose influenza virus infection [105,106]. Neutrophils, monocytes, and macrophages inhibit the spread of infection by phagocytosing infected apoptotic cells [107]. Neutrophils rapidly migrate to the site of infection and initiate phagocytosis, degranulation, and the formation of neutrophil extracellular traps [108]. The importance of neutrophils has been demonstrated in animal studies showing that neutrophil depletion leads to increased influenza mortality due to impaired viral clearance [109,110]. Neutropenic mice display reduced flu-specific CD8^+^ T cell responses in the respiratory tract [111]. Further to this, neutrophils guide CD8^+^ T cells to the infection site by leaving a trail of CXCL12 [112]. These studies indicate that neutrophils play a protective role in influenza infection, but that excessive recruitment of neutrophils can also contribute to immunopathology [113]. 

Alveolar macrophages (AMs) are lung-resident macrophages that control lung homeostasis at steady state and phagocytose opsonized pathogens and infected cells [114]. AMs are also the main producer of type I IFN in the lung during infection with RNA viruses [115]. There are several lines of evidence supporting the protective role of AMs during influenza infection. Clodronate-induced depletion of AMs increases the mortality of mice and enhances pulmonary inflammation during influenza infection [116]. *Csf2*^−/^^−^ mice, which are deficient in AM development, experience respiratory failure and reduced resistance to influenza infection despite having normal adaptive immune responses against influenza virus [117]. Consistently, an AM depletion model in CD169-DTR mice led to aggressive inflammatory responses and severe lung pathology after influenza infection [118]. AMs control lung dysfunction by increasing the antiviral resistance of type-1 alveolar epithelial cells, which are responsible for gas exchange [119]. These results indicate that AMs play a crucial role in maintaining pulmonary homeostasis during influenza infection.

### 4.2. Dendritic Cells

**Figure 3:**
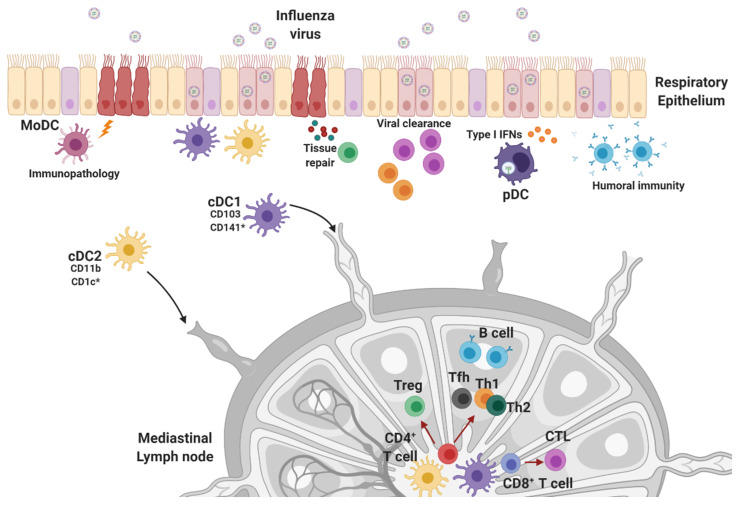
Overview of dendritic cell subsets in the lung and DC-mediated immune responses. After influenza virus infection, viral antigen-captured cDCs migrate to lymph nodes draining from the lung where they promote the activation of adaptive immune responses via antigen presentation. Both cDC1s and cDC2s have the capacity to induce CD4^+^ and CD8^+^ T cell activation, but cDC1s are generally characterized as cross-presenting DCs. pDCs, which produce type I IFNs, play a minor role in anti-influenza virus responses, and MoDCs are associated with immunopathology. *Human-specific marker.

DCs, which belong to the innate immune response system, are known as professional antigen-presenting cells. Because T cells only recognize foreign antigens that are associated with MHC molecules, DCs play a critical role in the priming and activation of naïve T cells. The specialized antigen-presenting function of DCs allows them to serve as the link between innate and adaptive immunity (Figure 3). Many studies have been conducted to clarify the role of DCs during influenza virus infection. CD11c-DTR transgenic mice express a high-affinity diphtheria toxin receptor (DTR) in CD11c^+^ cells [120], allowing targeted depletion of DCs by the administration of diphtheria toxin (DT). CD11c-DTR mice have been used to study the function of DCs in vivo. Following influenza virus infection, DT-treated mice experience significantly more severe weight loss than controls with intact DCs [121]. Further to this, in DC-depleted mice, influenza-specific CD8^+^ T cell populations are significantly decreased in the lung, and viral clearance is impaired, suggesting that CD11c^+^ DCs are responsible for the activation of CD8^+^ T cells and T cell-mediated viral clearance during influenza virus infection.

In the lungs, DCs are classified into migratory conventional DCs (cDCs), and pDCs at a steady state, and monocyte-derived DCs (MoDCs) are produced after exposure to inflammatory stimuli [122,123,124]. CD11c^hi^-MHC class II^+^ cDCs are distinguished by the surface expression of markers CD103^+^ migratory cDC1, and CD11b^+^ migratory cDC2 in mice, and CD141^+^ (BDCA-3), cDC1, and CD1c^+^ (BDCA-1), cDC2 in humans [125]. Several studies have suggested that the various DC subsets have specialized functions in the response to influenza virus infection. Remarkably, respiratory DC subsets display different susceptibility to influenza virus infection. cDC1 is more susceptible to influenza virus infection than cDC2 and pDCs [126], but influenza infection of cDC1 does not lead to productive viral replication [127]. Following influenza infection, lung-migratory cDCs take up viral antigen and migrate to the draining mediastinal lymph nodes (mLNs) where they present antigens to naïve T cells and induce adaptive immune responses. In the mLNs, migratory and resident cDCs can both prime T cells, and are subdivided into CD8^+^ cDC1 and CD11b^+^ cDC2. While resident CD8^+^ cDC1s in the mLNs have the potential to activate CD8^+^ T cells [128], mLN-resident cDCs seem to be less effective at inducing anti-influenza CD8^+^ T cell responses [127,129]. More research is needed to clarify the specific role of mLN-resident cDCs in the anti-influenza response.

Most studies determining the role of DCs in influenza virus infection have focused on the cDC1 subset. CD103^+^ cDC1s are well known as specialized cells that initiate CD8^+^ T cell activation via MHC class I-mediated cross-presentation of antigens. Lung-migratory cDC1s migrate into the mLNs to prime CD8^+^ T cells, and this process reaches its peak within 48–72 h after influenza virus infection [130]. There have been several reports that have suggested that CD103^+^ cDC1 is the dominant population for activation of effective CD8^+^ T cell responses against the influenza virus [121,127,131,132]. For example, Batf3^−/^^−^ mice, which lack cDC1s [133], cannot mount efficient anti-influenza virus CD8^+^ T cell responses [127,128]. Further to this, DT-treated Clec9A-DTR mice, which have been modified to express DTR in C-type lectin receptor-expressing cells such as cDC1, are highly susceptible to influenza virus infection after treatment with DT [129]. These cDC1-depleted mice were unable to recover from weight loss and had severe pulmonary inflammation and lung damage. In addition to reductions in effector CD8^+^ T cell function, the development of effective memory CD8^+^ T cell responses was severely impaired after cDC1 depletion. Indeed, cDC1s are involved in CD8^+^ T cell exit from the lymph node and CD8^+^ T cell survival. Taken together, cDC1s play a crucial role in enabling protective CD8^+^ T cell responses. Surprisingly, there is evidence supporting the capability of cDC1s to induce CD4^+^ T cell responses to IAV. In an in vitro T cell proliferation assay, cDC1s had superior CD4 and CD8 T cell expansion efficacy relative to pDCs and double-negative (CD8^-^ CD205^-^) DCs [132]. Furthermore, both human CD141^+^ cDC1 and CD1c^+^ cDC2 elicited CD4^+^ T cell proliferation and Th1 responses after stimulation with live-attenuated influenza virus [134], indicating that cDC1 can be responsible for both MHC class I- and class II-mediated antigen presentation.

cDC2 subsets are considered to be potent presenters of MHC-II-loaded antigens to CD4^+^ T cells [135]. While cDC1 subsets are the dominant DC population that transports antigens to lymph nodes that drain from the lung, cDC2s remain the dominant source of proinflammatory cytokines produced in the lung [131]. Despite the increased presence of cDC2s in lymph nodes that drain from the lungs during influenza virus infection, these cells rarely carried intact viral antigens [127]. Consistently, migratory cDC2s are less efficient at priming and expanding antigen-specific CD8^+^ T cells compared to migratory cDC1s [127,129]. However, cDC2s have the potential to cross-present antigens to CD8^+^ T cells [136], and a recent study demonstrated that IRF4-dependent DCs that express CD11b^+^CD24^hi^ contribute to the development of CD8^+^ T cell memory responses following IAV infection, suggesting that cDC2s promote the differentiation of memory CD8^+^ T cells [137]. Further investigation is required to better understand the role of cDC1s and cDC2s in the response to influenza virus infection.

pDCs, which are primarily known as producers of type I IFNs [138], seem to play a minor role in the defense against influenza virus infection. Despite the accumulation of pDCs in the lung following influenza virus infection, pDC-depleted mice showed identical survival rate and body weight loss compared to healthy controls [121,139]. Further to this, depletion of pDCs using the 120G8 antibody did not affect body weight loss, effector CD8^+^ T cell responses, or viral clearance after influenza virus infection in mice [121]. Consistently, both wild-type and pDC-deficient Ikaros^L/L^ mice showed comparable weight loss, viral burden in lung, influenza virus-specific antibody production, and lung pathology following viral infection [139]. However, there is some evidence that pDCs may cross-present influenza virus antigens to CD8^+^ T cells [140,141].

Although the role of MoDCs during influenza virus infection has not been well established, it has been reported that the number of CCR2-expressing MoDCs are increased in influenza virus-infected lungs [124]. These cells induce the robust production of nitric oxide synthase 2, and deficiency in CCR2 leads to attenuation in weight loss, pulmonary pathology, and mortality after a lethal dose of the influenza virus. While the specific mechanisms remain unknown, these results indicate that MoDCs are associated with influenza virus-induced pulmonary pathology. On the other hand, other work has suggested a protective role for MoDCs during secondary influenza virus infection [142]. Researchers found that MoDC-deficient CCR2^−/^^−^ mice displayed an impairment of antigen-specific CD8^+^ T cell formation following primary influenza challenge, and lack of MoDCs during primary influenza infection reduced host resistance to secondary virus infection, suggesting that MoDCs could be considered as putative targets of influenza vaccine. 

## 5. Protective Adaptive Immune Responses against Influenza Infections

Although innate immunity contributes to early viral control, adaptive immune responses are crucial for eventual viral clearance, recovery, and protection from reinfection. As expected, immunodeficient mice that are infected with influenza experience a significantly higher rate of mortality than healthy controls, confirming that adaptive immunity is crucial for the anti-influenza response [143,144]. In particular, the HA and NA proteins are primary targets of protective immunity.

### 5.1. CD4^+^ Helper T Cell Response

Naïve CD4^+^ cells recognize the viral antigens presented by the major histocompatibility complex (MHC) class II proteins on antigen-presenting cells, and subsequently differentiate into several types of helper T cells depending on the cytokine milieu. Activated CD4^+^ T cells support the activation and differentiation of antibody-producing B cells and also promote CD8^+^ T cells responses. CD4^+^ T cell responses peak at 10 days after influenza infection in the mouse lung [145]. Adaptive transfer of effector CD4^+^ T cells isolated from mice infected with influenza lead to increased survival of recipient mice after influenza challenge [145]. The cytokine milieu generated during influenza virus infection is polarized to support the generation of Th1 cells [146]. Th1 cells produce IFN-γ, IL-2, and tumor necrosis factor α (TNFα). These cytokines activate macrophages, promote B cells to produce IgG2a and IgG3 isotype antibodies [147], and mediate cellular immune responses. Th2 responses are also induced following influenza virus infection. Th2 cells secrete IL-4, IL-5, and IL-13 and promote isotype switching in B cells to produce other isotypes of antibodies such as IgG1 and IgE [146]. However, Th1 cells are more closely associated with survival after influenza virus infection compared with Th2 cells [148]. Some CD4^+^ T cell populations in influenza-infected mice show evidence of perforin/granzyme-mediated cytolytic activity [146,149,150]. In addition, IAV infection leads to robust regulatory T cells (Tregs) that mediate immunosuppression and tissue repair via IL-10 and amphiregulin [151,152], among other effector molecules. Notably, Treg depletion results in a reduction of the influenza-specific follicular helper T cell response [153]. These results indicate that Tregs play an important role in host protection during and after infection with the influenza virus.

### 5.2. Cytotoxic CD8^+^ T Cell Response

CD8^+^ T cells are important for viral clearance and host protection during influenza virus infection. CD8^+^ T cells recognize viral antigens loaded onto MHC class I proteins on the surface of viral antigen-presenting cells. CD8^+^ responses reach a maximum at 8 days after infection in the draining mLN and at 10 days post-infection in bronchoalveolar lavage fluid (BALF) [121,154]. β2-Microglobulin-deficient mice, which lack CD8^+^ T cells, display delayed viral clearance and severe mortality following influenza virus infection [155]. Activated cytotoxic CD8^+^ T cells (CTLs) eliminate virus-infected cells via cytolysis. They produce perforin to permeabilize the membranes of infected host cells and secrete granzyme into cells to induce apoptosis [156]. Additionally, CTLs can kill infected host cells via TNF receptor family-dependent pathways. CTLs express the Fas ligand (FasL) that binds to Fas on target cells, and the Fas–FasL interaction induces apoptosis via activation of a caspase cascade. TNF-related apoptosis-inducing ligand, also expressed on CD8^+^ T cells, is another mechanism for CD8^+^ T cell-mediated cytotoxicity [157]. Effector CD8^+^ T cells in the lung produce IFN-γ and TNFα, which contribute to viral defense mechanisms [158,159,160]. Remarkably, IL-10 is also produced by effector CD8^+^ T cells and is responsible for the regulation of pulmonary inflammation during the response to influenza virus infections. Previous studies have suggested that CD8^+^ effector cells are the major producers of IL-10 in the lungs of mice infected with the influenza virus [161]. Further, blockade of IL-10 signaling increases pulmonary inflammation and lethal injury following sublethal influenza virus challenge [161]. 

### 5.3. Humoral Immunity

The protective role of B cells in the anti-influenza virus immune response has been extensively studied in recent years. During influenza virus infection, naïve B cells in the mLNs encounter the influenza virus antigen and differentiate into antibody-forming cells (AFCs). B lymphocyte-deficient μMT mice are more susceptible to influenza virus infection compared to wild-type mice [162,163]. The B cell response against influenza virus begins approximately 3 days after infection, and B cells begin to secrete anti-influenza virus IgG by day 7 [164]. The total number of B cells in BALF peaks around 10 days post-infection in mice [121]. In mice, the majority of influenza virus antigen-specific AFCs in the lung produce IgG and IgM, but AFCs in the upper respiratory tract primarily produce IgA [165]. Systemic AFCs are first detected 6–7 days after infection. Antibodies specific for HA and NA are important for protective immunity because these proteins are responsible for viral entry and release. HA-specific antibodies bind to the HA globular head and inhibit the attachment of the virus to the host cell’s surface [166,167,168]. This mechanism of viral inactivation is called neutralization. NA-specific antibodies do not neutralize the virus, but instead block viral replication by inhibiting the enzymatic activity of NA [156,169]. M2 proteins are also the target of specific antibodies. Passive transfer of M2-specific antibodies provide protection against viral replication [170]. Surprisingly, NP-specific antibodies are protective in nature despite having an internal viral protein as their target [171]. In addition to the previously described functions, influenza virus-specific antibodies mediate antibody-dependent cell cytotoxicity and Fc receptor-mediated phagocytosis. Therefore, these antibodies also contribute significantly to the clearance of infected cells [172]. Moreover, B-1 cells that produce natural IgM, independent of antigenic priming, contribute to protection against influenza virus infection. Natural IgM in the airway has neutralizing activity and mediates early immune responses [173].

## 6. Conclusions

Influenza remains a major annual public health burden. Further, the average annual total economic burden of influenza has been estimated at $11.2 billion in the United States alone [174], and 294,000–518,000 deaths are caused by flu-related complications around the world each year [175]. Considerable efforts have been devoted to reducing the disease burden of influenza. Vaccination remains the best way to prevent influenza infection. Flu vaccines contain virus strains that are expected to circulate during the upcoming flu season, and vaccination can provide effective protection against viral infection. However, because influenza viruses constantly change their antigens to escape from vaccine-induced protection, we require a new flu vaccine each year. A long-standing goal of influenza research is the development of universal vaccines that provide protection against multiple subtypes of influenza viruses. Current studies are mainly focused on identifying conserved influenza epitopes, and the highly conserved HA stem has been suggested as one such promising target for universal vaccines [176,177]. Despite these encouraging recent developments, it is clear that further research is required to improve the effectiveness of universal vaccines. The current licensed influenza vaccine products have several limitations. Although the live-attenuated and inactivated vaccines can both confer virus-specific neutralizing antibody responses, only the live-attenuated vaccine induces cell-mediated immune responses [178]. 

Immunogenicity has been a major concern for influenza vaccine development. Novel types of vaccines, such as recombinant vaccines, DNA vaccines, and mRNA vaccines, are relatively poorly immunogenic [179], and several approaches have been tested to enhance their potency. For example, the inclusion of flagellin, a TLR5 agonist, has been shown to increase the effectiveness of recombinant influenza vaccines [180,181]. Similarly, the inclusion of a RIG-I agonist increases humoral immune responses to DNA influenza vaccines [182], suggesting that innate sensor agonists can improve the immunogenicity of new types of influenza vaccines. In order to overcome the limitations of traditional and emerging influenza vaccines, it is necessary to first understand the complex interplay between host immune responses and the pathologic mechanisms of influenza viruses.

The lack of proofreading ability in the viral RNA polymerase results in high mutation rates and the rapid evolution of influenza. The resulting changes in the protein targets of anti-influenza medications can lead to the rapid emergence of antiviral resistance. At present, there are three classes of FDA-approved influenza drugs targeting the influenza virus replication cycle. While these drugs are currently effective, a shifting pattern of changes in the viral genome can cause drug resistance and corresponding reductions in therapeutic efficacy. For example, most IAVs are now resistant to M2 channel inhibitors, and resistance to NA inhibitors has been reported with varying frequency across seasonal outbreaks [183,184,185,186]. Therefore, there is still a need for improved strategies for the prevention and treatment of influenza.

The current set of vaccines and anti-influenza drugs are effective at reducing the risk and severity of influenza infection. However, there is still room for improvement. Advancing our understanding of virus–host interactions will provide not only new fundamental information, but also valuable insights for the development of improved therapeutics.
